# Deciphering the role of crucial miRNAs involved in diabetic cardiomyopathy through a multiomics approach

**DOI:** 10.1038/s41598-025-09084-x

**Published:** 2025-07-08

**Authors:** Bhagyalakshmi Balakrishnan, Raghu Chandrashekar Hariharapura, Divyashree Somashekara, Fayaz Shaik Mahammad

**Affiliations:** 1 Department of Biotechnology, Manipal Institute of Technology, Manipal Academy of Higher Education, Manipal, 576104 India; 2 Department of Pharmaceutical Biotechnology, Manipal College of Pharmaceutical Sciences, Manipal Academy of Higher Education, Manipal, 576104 India

**Keywords:** Bioinformatics, miRNA, Diabetic cardiomyopathy, Network analysis, Hub genes

## Abstract

Conventional drug discovery routes take decades to identify critical gene signatures involved in a disease. Though in silico platforms and software have simplified the process by reducing the time and energy needed to arrive at significant molecular targets for disease conditions, they are not accessible to everyone. Here, we propose a systematic strategic protocol to identify critical players in the disease pathogenesis using the vast amount of molecular omics data available on various public domains, setting diabetic cardiomyopathy as the case study. We use databases like KEGG, DisGeNET, HMDD, and literature to curate the key genes and miRNAs associated with the disease. We analysed 395 genes and 20 miRNAs for our analysis. Critical pathways like the insulin resistance pathway, the AGE–RAGE pathway, and the PI3K–AKT–mTOR pathway were enriched in our analysis through the Metascape platform. A protein–protein interaction network was generated using the STRING database. The export of this network to the Cytoscape platform helped to identify the clusters, node interactions, and hub genes. Our approach finally identified hsa-miR-302a-3p as the critical miRNA that regulates the highest-ranked hub gene, *AKT1*. This systematic methodology lays a solid foundation for the identification of critical therapeutic targets for any disease condition, thereby increasing the success rate for further stages of development.

**Supplementary Information:**

The online version contains supplementary material available at 10.1038/s41598-025-09084-x.

## Introduction

Diabetic Cardiomyopathy (DCMY) was presented as a mysterious case in 1972 by Rubler et al., who found that four out of 27 diabetic patients’ postmortem results showed unusual cardiomegaly and congestive heart failure without any visible cause like atherosclerosis or hypertension. The clinical presentations included left or right ventricular hypertrophy, interstitial fibrosis, and thickening of the coronary arterial lumen due to the deposition of acid mucopolysaccharide^1^. DCMY gained more attention after the 1974 Framingham study, a multigenerational study dedicated to understanding the factors responsible for cardiovascular conditions^2^. Though there is no unified global survey on DCMY, the growing diabetic pandemic translates to an increased DCMY burden in society. According to the IDF Diabetes Atlas, an estimated 537 million people worldwide had diabetes in 2021. This number is projected to rise to 643 million by 2030^3^. And approximately 12% of individuals with diabetes go on to develop cardiomyopathy^4^. Given this scenario, research into DCMY is more critical than ever. Advancing our understanding of its mechanisms, developing early diagnostic biomarkers, and discovering novel treatments are essential to reducing morbidity and improving outcomes for this growing patient population.

Diabetic people have 3–5 times higher chances of heart failure than a non-diabetic person^2^. Cardiac complications and heart failure are identified as the leading cause of mortality among diabetic patients^5^. The molecular pathophysiology of DCMY escalates in parallel with type 2 diabetes mellitus (T2DM). Insulin resistance (IR) is considered as the root cause for diabetes, DCMY and many other metabolic disorders. IR affects the cellular ability to uptake glucose from the blood circulation^6^. IR also increases hepatic gluconeogenesis, further contributing to hyperglycemia^7^. These changes lead to glucotoxicity in cardiomyocytes. Davargaon et al. demonstrated that high glucose exposure in cardiomyocytes led to oxidative stress and mitochondrial dysfunction^8^. Wende et al. identified that glucotoxicity disrupts mitochondrial oxidative phosphorylation through post-translational modifications, reducing energy efficiency. These alterations culminate in the inability of the heart to adjust to the metabolic sources of fuel according to the physiological conditions. This loss of metabolic plasticity of the heart is an essential hallmark of diabetic cardiac dysfunction^9^. Again, loss of metabolic plasticity results in lipotoxicity and more oxidative stress.

While discussing the clinical presentations, left ventricular dysfunction (LVDD), hypervascular and interstitial fibrosis, cardiac remodeling, and ultimately heart failure are the critical features of the condition^2,10,11^. LVDD is one of the initial indicators of the disease and ultrasound echocardiography can detect this to an extent^4^. Cardiac wall thickening and interstitial fibrosis affect the normal functioning of the heart. There is reduced left ventricular ejection fraction and reduced pumping of blood by the heart.

Though many genes are implicated in the above-discussed pathways that link metabolism, inflammation, and even the body’s immune system to DCMY, these would only be a few examples of the genetic predispositions of DCMY, which have been the subject of recent research. More linkage studies and genome-wide association studies are required to explore the genetics of the illness fully. Although genetic susceptibility plays a significant role, poor lifestyle or dietary choices can influence or worsen DCMY, proving the epigenetic basis for the condition.

miRNAs are particularly known to be the crucial regulators of epigenesis. Emerging evidence underscores the pivotal role of miRNAs in the pathogenesis of DCMY, where they regulate gene expression post-transcriptionally, influencing processes such as fibrosis, apoptosis, and oxidative stress. Recent studies have employed integrated bioinformatic analyses to identify differentially expressed miRNAs and their target genes in DCMY. A study by Zhou et al. conducted an integrated bioinformatics analysis to identify regulatory networks involved in disease development^12^. Yu et al. (2022) demonstrated that overexpression of miR-92a-2-5p ameliorated oxidative stress-induced injury in cardiomyocytes, suggesting its role in mitigating DCMY progression^13^. Similarly, inhibition of miR-92a has been shown to enhance angiogenesis in cardiac microvascular endothelial cells from diabetic patients^14^. Furthermore, mesenchymal stem cells have been found to alleviate inflammation and pyroptosis in DCMY via the miR-223-3p/NLRP3 pathway^15^. These findings collectively emphasize miRNAs’ significance as biomarkers and therapeutic targets in DCMY, warranting further exploration through integrated bioinformatic analyses. These findings suggest that miRNAs serve as biomarkers for early detection and potential therapeutic targets.

This kind of system biology approach incorporates various omics layers of biomolecules from different classes, such as genes, proteins, and non-coding RNAs, allowing the exploration of disease pathophysiology in detail. Given the multifaceted aspects of DCMY, a multi-omics framework is crucial for advancing personalized medicine and optimizing targeted interventions. In this study, the regulatory networks between the key miRNAs, their target genes, and the pathways related to DCMY were constructed and analyzed. This integrated bioinformatic approach would aid in the exploration of significant miRNAs as biomarkers and therapeutic targets in DCMY.

## Results

### Genes associated with DCMY and expression analysis

395 genes associated with DCMY were curated from various databases for this study. Kyoto Encyclopedia of Genes and Genomes (KEGG) disease database exhibited 203 genes experimentally proven to be dysregulated in DCMY^16^. The DisgeNET v24.0 database showed 220 genes associated with DCMY^17^. Out of the 395 genes from these two databases, 27 shared genes were significantly differentially expressed with Padj < 0.05 and Log2FC > 1.5 (Supplementary Table S1). These 27 shared genes were considered for further investigation, to increase the confidence of analysis across independent studies.

The NCBI Gene Expression Omnibus (GEO) database was explored to investigate the expression profile of the 395 genes in DCMY^18^. The dataset GSE197850, based on the Illumina HiSeq 2000 (Homo sapiens) was used to identify the differentially expressed genes (DEGs) in DCMY compared to control through the GEO2R interface. Quality control plots like boxplots, volcano plot, and a UMAP plot were generated for the identification of biologically relevant DEGs for subsequent analyses (Supplementary Figures S2, S3, and S4).

The GEO2R platform identified the regulation of 188 genes from KEGG, of which 59 genes were found to be downregulated, and 129 genes were upregulated. The 220 genes from DisGeNET were also similarly analysed. Out of the expression profile of 156 genes, identified through the GEO platform, 76 were downregulated, and 80 were upregulated. Upregulated genes from the KEGG database were intersected by with those from DisGeNET by Venn analysis^19^ to identify commonly upregulated genes. A total of 14 genes (RYR2, ACE, CD36, AGT, AGER, VDAC1, PLN, PPARA, PIK3CD, TGFB1, ATP2A2, PTPA, MAPK14, and PRKCD) were found to be common to both platforms (Supplementary Figure S5a). A similar strategy was employed for the downregulated gene subset. Thirteen genes (SLC25A4, TNNI3, NOS3, SMAD2, PIK3CA, MAPK8, AKT1, GSK3B, PARP1, PIK3CB, REN, INS and SLC2A1) were found to be overlapping among the two databases (Supplementary Figure S5b). Finally, the 27 differentially expressed shared genes (14 upregulated and 13 downregulated) were considered for further investigation.

### Pathway enrichment in DCMY

**Fig. 1:**
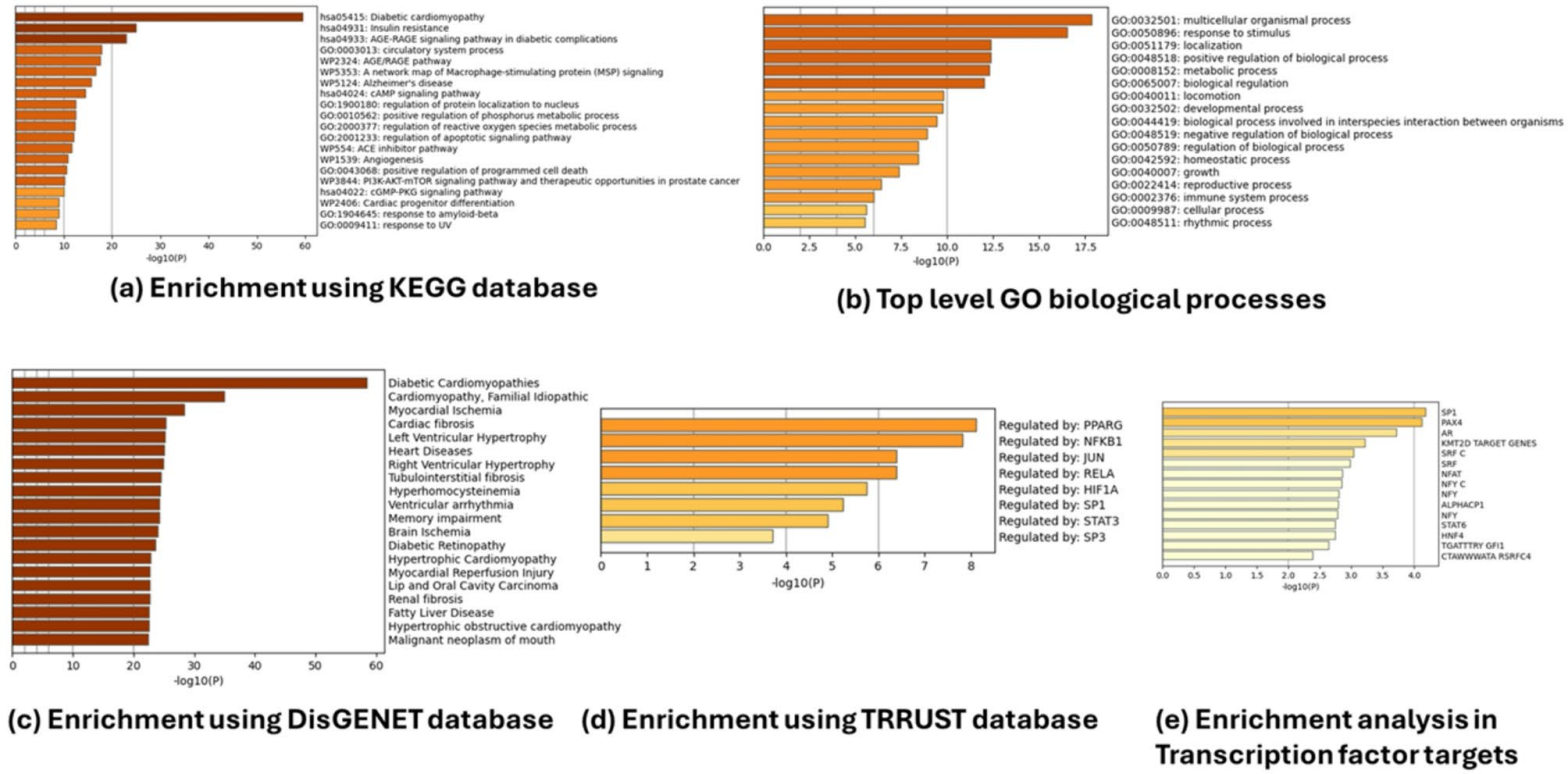
Enrichment analysis of the 27 genes associated with DCMY using KEGG and GO libraries in Metascape (**a**) Bar graph representing enriched pathways, colored according to p-values (**b**) Enriched GO biological processes (**c**) Enrichment Analysis in DisGeNET (**d**) Enrichment Analysis in TRRUST (**e**) Enrichment Analysis in Transcription Factor Targets.

The enrichment analysis of these 27 genes was carried out using the KEGG pathway database. DCMY related genes were highly linked to insulin resistance pathway, AGE–RAGE pathway, macrophage-stimulating protein (MSP) signaling and the ACE inhibitor pathways as shown in Fig. 1a–e. Furthermore, other relevant signaling pathways that are enriched in the gene set were the regulation of reactive oxygen species metabolic process, cAMP signaling pathway, PI3K–AKT–mTOR signaling pathway, cGMP-PKG signaling pathway, regulation of apoptotic signaling pathway, and cardiac progenitor differentiation pathways (Fig. 1a). Gene Ontology (GO) biological processes revealed the involvement of these genes in metabolic and developmental processes, among other basal biological functions (Fig. 1b).

The Metascape enrichment analysis^20^ using the DisGeNET database identified that apart from DCMY, the genes are also linked to other cardiomyopathic types, especially Familial idiopathic cardiomyopathy, myocardial ischemia, cardiac fibrosis, and ventricular hypertrophy (Fig. 1c). TRRUST (transcriptional regulatory relationships unraveled by sentence-based text-mining) database analysed our dataset with transcriptional regulatory networks and showed that PPARG and NFKB1 are highly involved in DCMY. Transcriptional factors like JUN and RELA showed moderately high gene set regulation along with HIF1A, SP1, STAT3, and SP3 (Fig. 1d). SP1 and PAX4 were highly represented among the transcription factor targets (Fig. 1e).

### Protein–protein interaction clusters

**Fig. 2:**
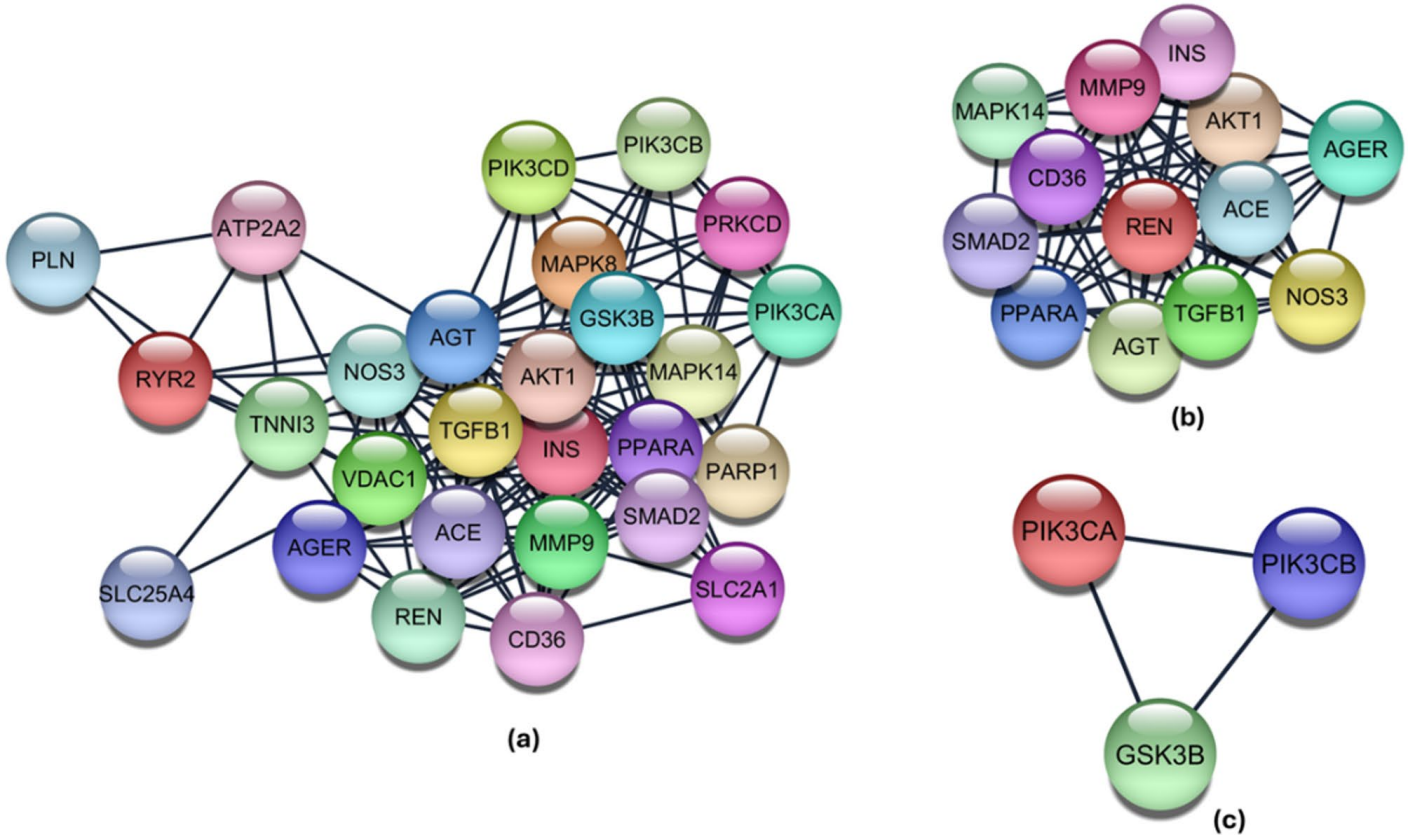
The stringified PPI network (**a**) The 27 shared genes related to DCMY across public databases (**b**) Cluster 1 by MCODE Cluster analysis (**c**) Cluster 2 by MCODE Cluster analysis.

The protein–protein interaction (PPI) network generated from STRING^21^ had 27 nodes and 151 edges. The average node degree was 11.2. The average local clustering coefficient was 0.671 and the enrichment p-value of the network was < 1.0e-16. The confidence cut-off taken here was 0.7 (Fig. 2a). The PPI network analysed using Cytoscape v3.10.2 platform^22^ with the MCODE plug-in detected two prominent clusters (Fig. 2b,c) (Supplementary Table S2). The gene name, node status, and MCODE score for each cluster are given in Supplementary Tables S3 and S4.

**Fig. 3:**
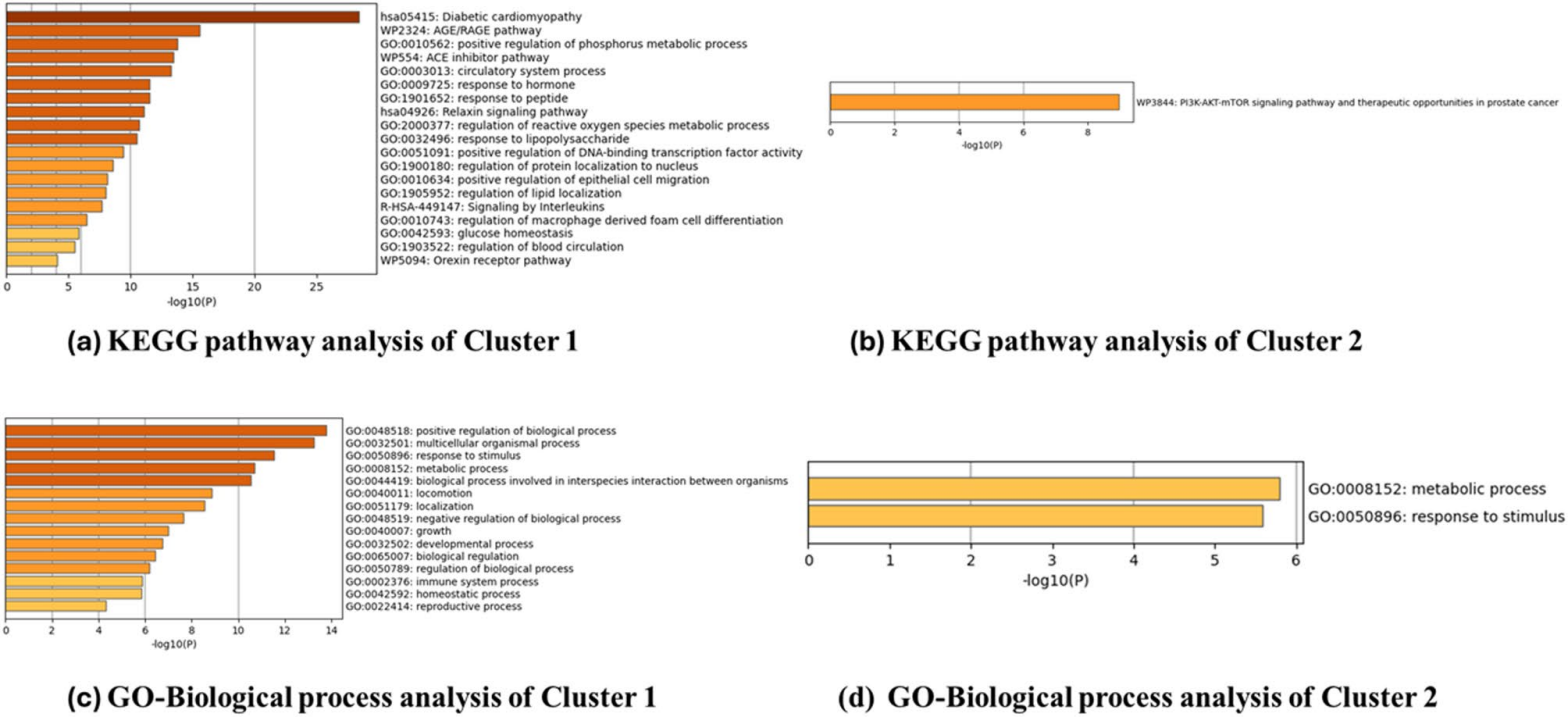
Analysis using Metascape. (**a**) KEGG pathway analysis of Cluster 1 (**b**) KEGG pathway analysis of Cluster 2 (**c**) GO-Biological process analysis of Cluster 1 (**d**) GO-Biological process analysis of Cluster 2.

Pathway analysis was also performed on these clusters separately, using the KEGG database (Fig. 3a,b) and GO analysis (Fig. 3c,d). The Cluster 1 proteins were involved in the AGE–RAGE pathway significantly alongside the classical diabetic cardiomyopathy pathway. There was also significant representation of the positive regulation of the phosphorus metabolic process, the ACE inhibitor pathway, the relaxin signaling pathway, and the reactive oxygen species metabolic process (Fig. 3a). Cluster 2 showed high annotation of the PI3K–AKT–mTOR pathway (Fig. 3b). The GO biological processes majorly demonstrated a positive regulation of biological processes, metabolic processes, and response to stimuli, indicating that DCMY is a metabolic disorder (Fig. 3c,d).

### PPI network topological parameters and hub genes

**Fig. 4:**
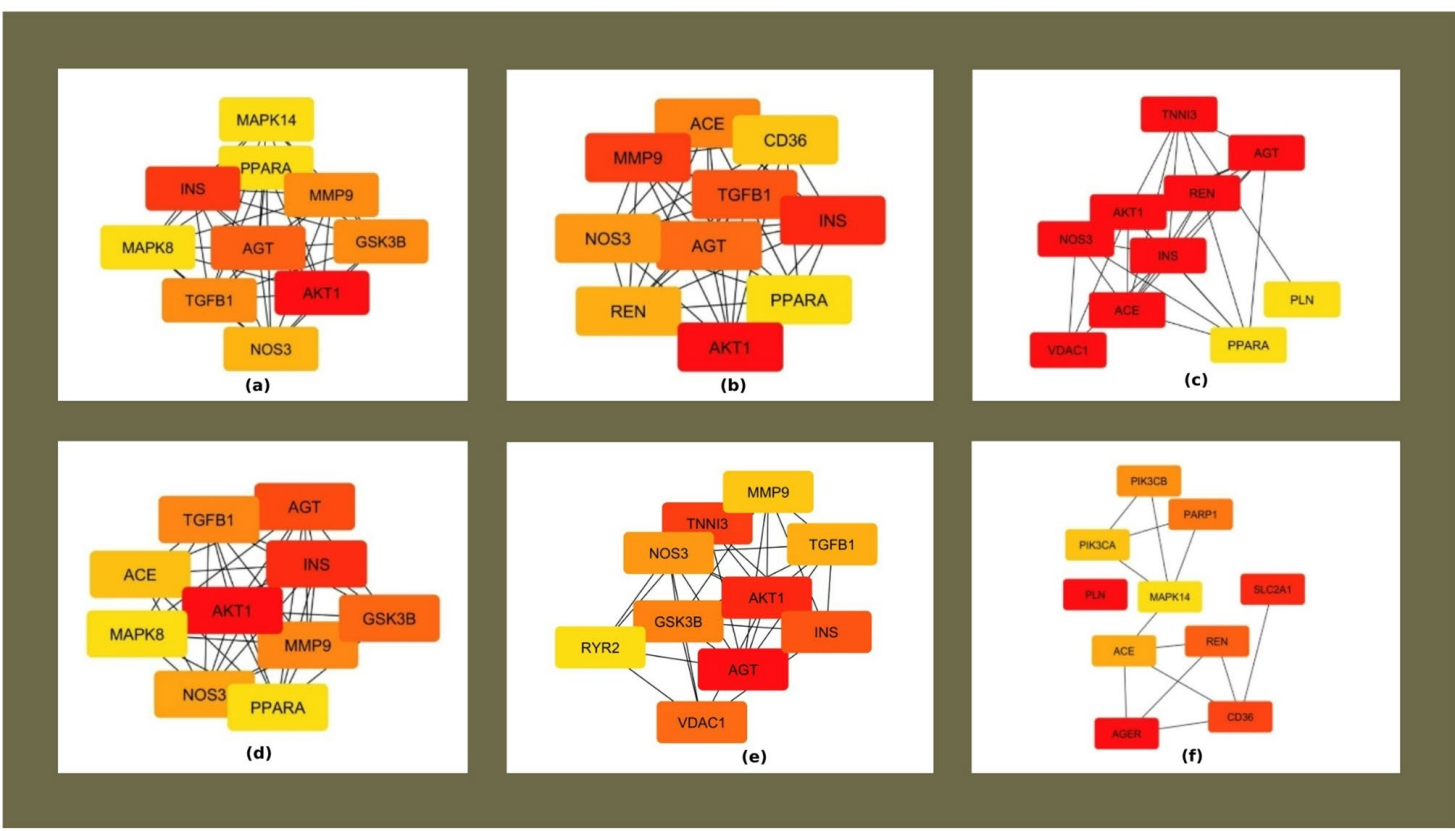
Cytohubba analysis of the 27 genes associated with DCMY (**a**) betweenness ranking method, (**b**) degree ranking method, (**c**) eccentricity ranking method, (**d**) MCC ranking method, (**e**) closeness ranking method, and (**f**) clustering coefficient ranking method. The red to yellow color gradient represents the hub gene ranking from high to low.

The crucial genes in the PPI network were assessed based on various topological parameters like the betweenness, degree, eccentricity, Maximal Clique Centrality (MCC), closeness, and clustering coefficient ranking methods (Fig. 4a–f).

AKT1 was interestingly ranked high in four measures: MCC, closeness, degree, and eccentricity. AGT was top ranked by the parameters of betweenness and eccentricity. PLN and AGER were ranked high by the Clustering coefficient parameter. The Eccentricity feature strikingly ranked AGT, AKT1, TNNI3, INS, VDAC1, NOS3, REN, and ACE with a score of one. Genes ranked according to various parameters by Cytohubba are given in Table 1.

**Table 1:**
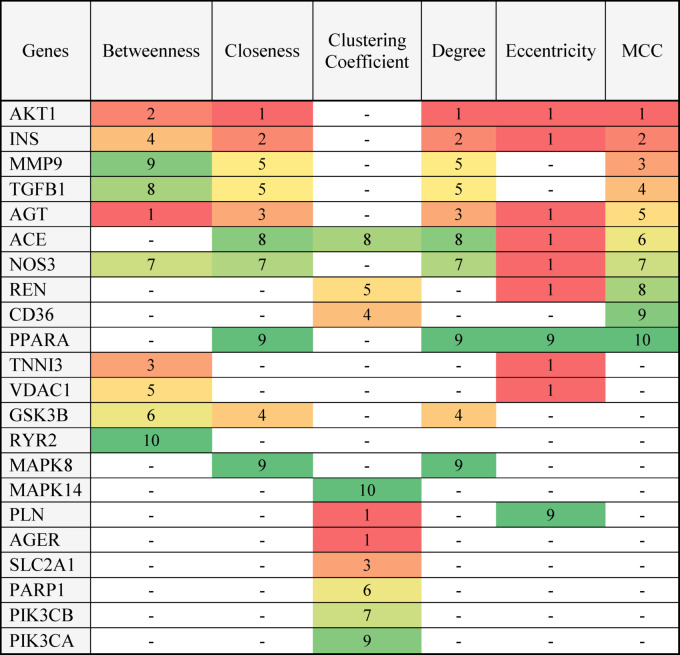
The ranks of the hub genes as analyzed by Cytohubba based on six different parameters.

*Ranks were assigned using six Cytohubba topological measures. Ranks are color-coded: red = highest rank (most central); green = lowest rank.

### Common miRNAs in DCMY

The Human MicroRNA Disease Database (HMDD) version 4.0 and literature survey provided the miRNAs associated with DCMY. Sixteen miRNAs were identified from the HMDD database, which were experimentally proven to be differentially regulated in DCMY. The study by Lu et al. (2010) reported four miRNAs, hsa-miR-34, hsa-miR-214, hsa-miR-223, and hsa-miR-302a-3p to be crucial in DCMY. The regulatory roles of these 20 miRNAs in DCMY considered for this study were confirmed through literature survey^23,24^ (Supplementary Table S5).

### Functional profiling of the miRNAs

The functional profiling of the 20 miRNAs was carried out using Metascape server. MiRNA-mediated post-transcriptional gene silencing was the highly enriched pathway (GO:0,035,195) (Supplementary Figure S1). MirPath v4 database analysis using the GO library was performed on the miRNAs associated with DCMY^25^. The GO analysis for biological processes using the classical method identified that these miRNAs majorly regulate pathways related to the positive regulation of transcription by RNA polymerase II. Signaling pathways like phosphatidylinositol 3-kinase signaling, cytokine-mediated signaling, and protein kinase B signaling were also enriched with significant p-values (Table 2).

**Table 2:** The pathways and target genes of miRNAs identified from the MirPath database.

Pathway	No. of genes	Target genes (n)	miRNAs (n)	P-value
Positive regulation of transcription by RNA polymerase II	1,258	149	24	3.71E-52
Cytokine-mediated signaling pathway	319	69	23	2.44E-40
Positive regulation of phosphatidylinositol 3-kinase signaling	96	29	17	6.27E-22
Positive regulation of protein kinase B signaling	188	43	22	1.79E-26

### Curation of miRNA targets specific to DCMY

**Fig. 5:**
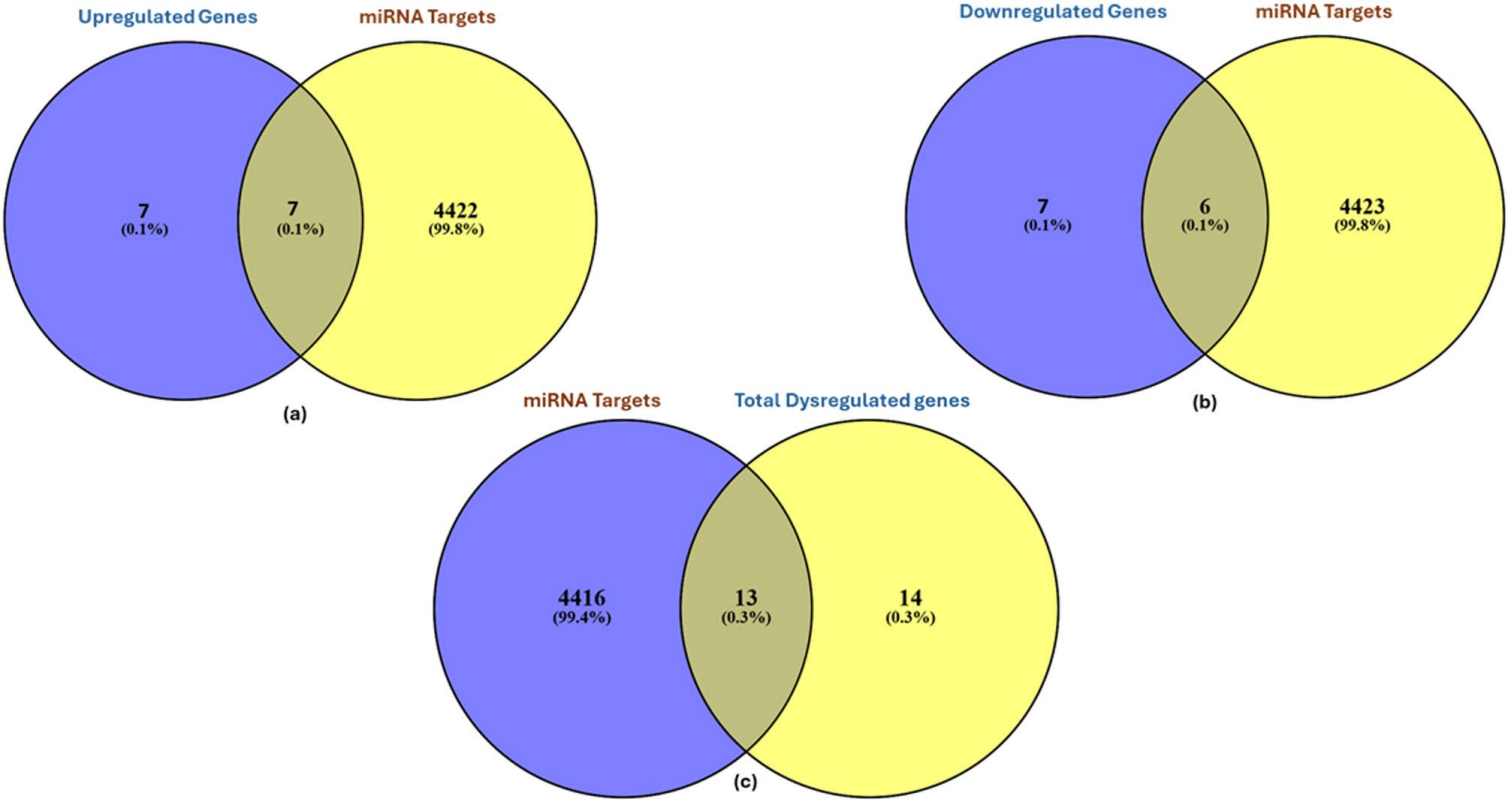
Venn Diagram to find the intersection of the miRNA targets and the genes of DCMY. (**a**) intersection of the miRNA targets and the upregulated genes of DCMY (**b**) intersection of the miRNA targets and the downregulated genes of DCMY (**c**) intersection of the miRNA targets and the total dysregulated genes of DCMY.

The miRTarbase 9.0 database^26^ provided the targets of 16 out of the 20 miRNAs, which included 4429 miRNA-target interactions (MTIs). Seven genes (*AGER, PPARA, TGFB1, ATP2A2, MAPK14, MMP9 and PRKCD*) were found to be common among the upregulated genes and miRNA targets (Fig. 5a). Six genes (*SMAD2, MAPK8, AKT1, GSK3B, PARP1, and SLC2A1*) were prominently found to be shared between the downregulated genes and miRNA targets (Fig. 5b). A total of thirteen genes (*ATP2A2, GSK3B, MAPK14, PRKCD, SMAD2, AGER, MAPK8, TGFB1, SLC2A1, PARP1, AKT1, PPARA, and MMP9*) were identified to be common between the miRNAs target genes and the 27 shared genes of DCMY (Fig. 5c).

### The miRNA-gene-pathway network

**Fig. 6:**
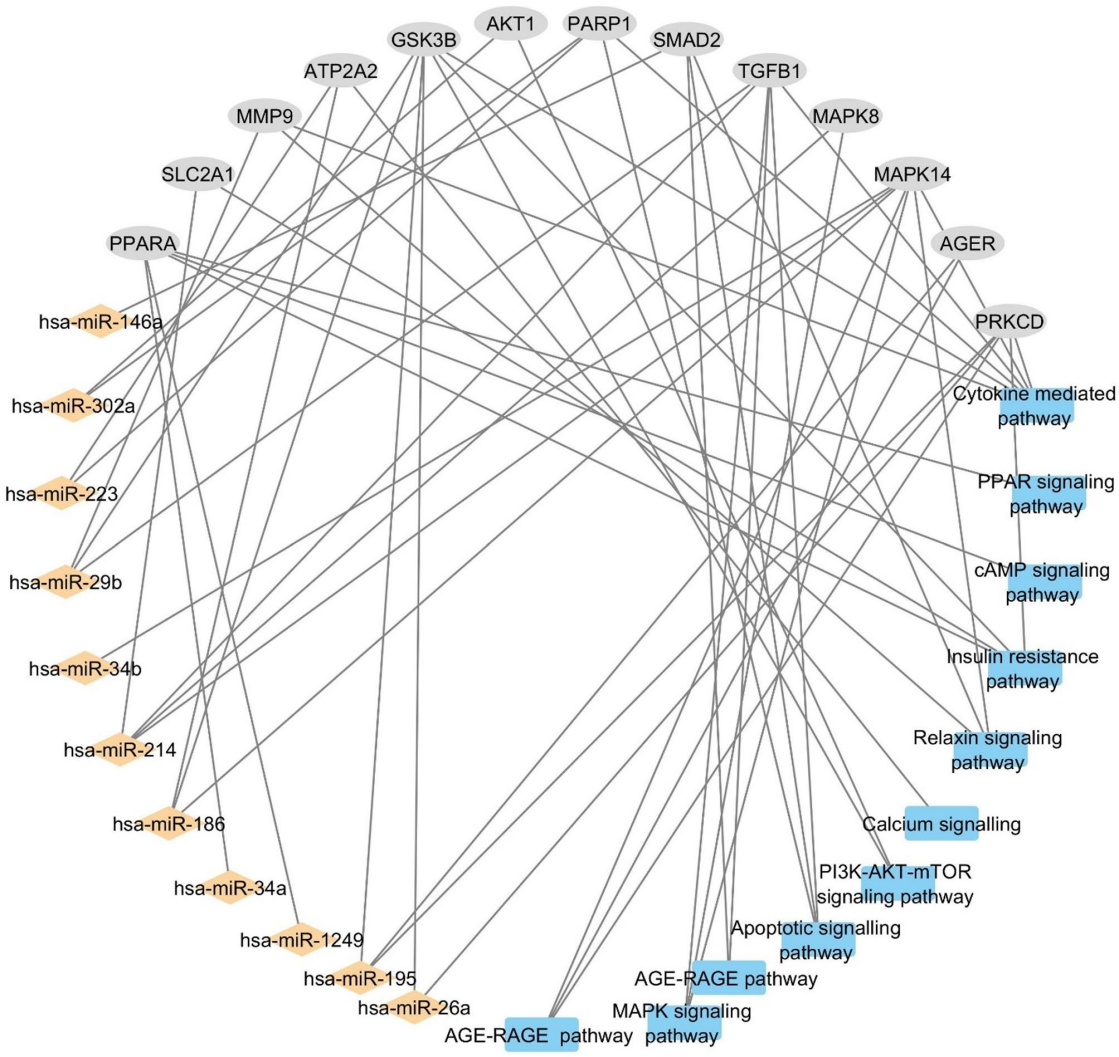
miRNA-gene-pathway network was manually generated using Cytoscape with the miRNAs, DCMY gene targets, and associated pathways. Orange filled diamond represent the miRNAs, grey filled oval circle represent the genes, and blue filled rectangle represent the pathways.

The network of miRNAs, their target DCMY genes and the pathways involving these genes was constructed using Cytoscape (Fig. 6). The network consists of 11 miRNAs indicated by the orange diamond-shaped nodes, and the 13 DCMY genes indicated as blue-coloured rectangular nodes. The miRNA hsa-miR-214-3p was found to target four genes, namely *MAPK14, MAPK8, SLCA1* and *TGFB1*.Similarly, hsa-miR-29b-3p targeted *TGFB1, MMP9* and *GSK3B*. hsa-miR-195-5p targeted *AGER, PRKCD*, and *GSK3B*. hsa-miR-186-5p targeted *ATP2A2, MAPK14* and *GSK3B*. hsa-miR-34b-3p was found to regulate *MAPK14*. hsa-miR-223-3p targeted *PARP1* and *ATP2A2*. hsa-miR-34a-5p and hsa-miR-1249-5p were found to target *PPARA.* Interestingly, hsa-miR-302a-3p targeted *AKT1* and *PARP1. AKT1* is the most highly ranked hub gene. Apart from *AKT1,* this miRNA was also found to regulate *PARP1.*

## Discussion

Systems biology and bioinformatics help integrate molecular data and provide a vibrant picture of the disease mechanism. High-throughput data containing disease-related genes or miRNAs from real-life diagnostic or routine clinical settings are far more available today. This clinical data-driven approach is highly reliable and more associable with human pathology than in vitro or in vivo disease models.

Here, we chose Diabetic Cardiomyopathy as our case study. The differentially regulated miRNAs in DCMY were collected from public databases and published literature. This data was then integrated to identify the critical miRNAs through network analysis. These miRNAs could be potential biomarker candidates for DCMY diagnosis, or a therapeutic for better prognosis.

We first pooled the genes related to DCMY from various databases. The shared differentially expressed genes among the databases were then selected for the study. The enrichment analysis of the shared DCMY-related genes across public databases revealed a high association with the insulin resistance pathway (Fig. 1a). The insulin signaling pathway is believed to be the classical system for glucose homeostasis in all metazoans^27^. Insulin receptor tyrosine kinase regulates glucose homeostasis in the cell based on its interaction with insulin. The signal is relayed mainly through the canonical PI3K–AKT–mTOR pathway. This pathway was significantly represented in the second cluster of the PPI network (Fig. 2c). Hyper-activated insulin signaling systems cause IR, the principal complication behind many metabolic disorders. IR is sustained through the overactive mTOR pathway, the primary feedback system of the insulin signaling pathway^28^. Various cell types show IR, like skeletal myocytes, cardiomyocytes, hepatocytes, and adipocytes.

In addition to IR, the DCMY genes were highly mapped to the AGE–RAGE pathway. Hyperglycemia leads to excessive superoxide radical generation in mitochondria and oxidative stress. This causes irreversible modifications to biomolecules like proteins, nucleic acids, and lipids, known as Advanced Glycation End-products (AGEs). RAGE is a pattern recognition receptor that binds AGE ligands. Various AGEs binding to RAGE stimulate different signaling cascades^29^. These facts showcase the significance of RAGE as an explicit target for obliterating DCMY. The interaction of AGE and RAGE activates NFKB1, the indispensable driver of systemic inflammation.

Another interesting pathway that was annotated in the Metascape analysis was the MSP signalling pathway. This is significant because MSP regulates macrophage activity and modulates fibrosis, supporting its value as a candidate for therapeutic investigation^30^. Similarly, the ACE inhibitor pathway was observed in the analysis. ACE inhibitors help reduce the detrimental effects of the Renin-Angiotensin-System-mediated inflammation, fibrosis, and remodelling^31,32^.

The DisGeNET database-based investigation showed that, though the genes in the study are specific for DCMY, they are also related to familial idiopathic cardiomyopathy (ICM), myocardial ischemia (MI), and cardiac fibrosis, among others (Fig. 1c). Abnormalities of ventricular wall thickness, size of the ventricular cavity, contraction, relaxation, conduction, and rhythm are the characteristics of ICM^33^, which are similar to DCMY characteristics. MI without significant atherosclerosis or coronary artery disease is an overlooked phenomenon^34^. The structural and functional abnormalities of the myocardium could be due to cardiac remodeling as a result of hyperglycemia and glucotoxicity. Cardiac fibrosis is another clinical manifestation of pathological extracellular matrix (ECM) remodeling. There are abnormalities in matrix composition and quality affecting the functional abilities of the myocardium^35^.

The TRRUST database, which curates transcriptional regulatory relationships between transcription factors and their target genes, showed the over-representation of NFKB1 (Nuclear Factor Kappa B Subunit 1) and PPARG (peroxisome proliferator-activated receptor gamma) in our data (Fig. 1d). PPARs are nuclear receptors that form heterodimers with retinoid X receptors and regulate transcription of various genes. Notably, PPARG is a regulator of adipocyte differentiation and has been implicated in the pathology of diabetes.

The 27 genes associated with DCMY were used to construct a PPI network, and clustering was performed on this network. MCODE clustering analysis gave 2 clusters. Cluster 1 had the nodes PPARA, AGER, ACE, REN, CD36, NOS3, MAPK14, SMAD2, TGFB1, AKT1, MMP9, INS, and AGT. The seed node identified was PPARA. GO-biological process analysis showed that the genes in Cluster 1 (see Supplementary Table S3 online) were mainly involved in the positive regulation of biological processes, multicellular organismal processes, response to stimulus, and metabolic processes. Cluster 2 had the nodes PIK3CA, GSK3B, and PIK3CB. This cluster was enriched with nodes involved in metabolic processes and response to stimuli.

These 27 genes were also analysed using the Cytohubba plugin in Cytoscape. The PPI network was analyzed mainly using six topological measures: betweenness, closeness, clustering coefficient, degree, eccentricity, and MCC. These topological measures enhance the biological relevance of identified hub genes by capturing diverse aspects of node centrality and network connectivity. While degree centrality highlights highly connected nodes, betweenness and closeness emphasize nodes critical for information flow and network communication. The clustering coefficient assesses the local interconnectedness of a node’s neighborhood, and eccentricity reflects a node’s relative position within the network. MCC further refines hub detection by identifying nodes involved in highly interconnected subnetworks. These six parameters were selectively chosen from the broader set available in CytoHubba based on their complementary nature and interpretability for robust hub gene identification. They collectively balance global and local network properties, making them more biologically meaningful than other less informative or redundant metrics. The Cytohubba analysis of the 27 shared genes showed *AKT1* to be the highly ranked gene on many topological measures. The genes *AGT, PLN,* and *AGER* also scored high in a few parameters.

*AKT1* emerged as a prominent hub gene in the network analysis, ranking highest in closeness, degree, eccentricity, and MCC scores. This consistent topological centrality across multiple complementary parameters underscores its critical role in maintaining network integrity and mediating signal transduction. Its high degree indicates extensive connectivity with other nodes, suggesting involvement in multiple regulatory interactions. The elevated closeness centrality reflects *AKT1*’s efficiency in communicating with all other nodes, while its top eccentricity score points to a central positional advantage within the network structure. Furthermore, its leading MCC value indicates participation in densely interconnected subnetworks, highlighting its potential role in coordinating key functional modules. This proves the biological importance of *AKT1* as a central regulatory node and potential therapeutic target within the studied molecular framework.

The miRNAs involved in DCMY were identified from the HMDD v4.0 database and literature survey. The HMDD v4.0 contains over 53,000 entries, including recent categories such as exosomal miRNAs, virus-encoded miRNAs, and miRNA-circRNA interactions, as well as information on sex-biased miRNAs in disease contexts. It serves as a valuable tool for researchers investigating the roles of miRNAs in disease pathogenesis, diagnosis, and therapy, providing a robust foundation for hypothesis generation and validation in miRNA-related biomedical studies. Literature was also intensively searched for original articles to get high-throughput information on miRNA or gene profiles for a comparative analysis to infer the differentially expressed genes in DCMY versus control. Lu et al. (2010) worked on miRNA profiling from left ventricular biopsies of patients with LVDD with or without T2DM. The patients were awaiting routine cardiac surgery, and the study followed the principles of the Declaration of Helsinki. Four miRNAs that were differentially regulated were selected from this study^22^. The regulatory roles of these miRNAs were also studied through literature surveys. Yang et al. (2024) demonstrated that mesenchymal stem cells alleviate inflammation and pyroptosis in diabetic cardiomyopathy by modulating the miRNA-223-3p/NLRP3 signaling pathway, suggesting a potential therapeutic mechanism^15^. Zhu et al. (2017) investigated the miR-34a-5p/Sirt1/p66shc axis in doxorubicin-induced cardiotoxicity^36^. Qi et al. (2020) elucidated the LncRNA-MIAT-mediated miR-214-3p silencing associated with cardiac fibrosis^37^. Tang et al. (2018) reported that hsa-miR-302a-3p may inhibit epithelial-mesenchymal transition in diabetic kidney disease by targeting ZEB1, suggesting its broader role in diabetic complications^38^. Furthermore, Samidurai et al.(2020) established the cardioprotective role of hsa-miR-302a-3p in myocardial reperfusion injury^39^.

The targets of these miRNAs were curated from miRTarBase database. The functional profiling of these targets revealed that they are mainly transcriptional activators. The GO pathway analysis identified these miRNAs to be involved in PI3K signaling, protein kinase B (AKT signaling). miRNA-mediated post-transcriptional gene silencing and negative regulation of biological processes are also seen. This increases the significance and reliability of our annotated miRNAs because the PI3K–AKT–mTOR pathway plays a central role in cardiomyocyte survival, hypertrophy, and metabolism. The dysregulation is linked to cardiac remodeling and progression of heart failure, hallmark features of DCMY. DCMY also involves chronic inflammation and immune system activation. The cytokine-mediated signaling pathway mediates intercellular communication in inflammatory responses and contributes to cardiac tissue damage and fibrosis. Thus, miRNAs regulating these pathways are of immense therapeutic potential.

The thousands of targets of these miRNAs were then compared with the 27 shared genes of DCMY. Eleven gene targets were deciphered from the Venny intersection analysis. These targets and their miRNAs were integrated into Cytoscape to generate the miRNA-gene network. However, not all these genes were high-ranked hub genes. Meanwhile, hsa-miR-302a-3p was the only miRNA that regulated *AKT1*, the most highly ranked hub gene.

The miRNA hsa-miR-302a-3p has also been previously reported to be involved in regulating microvesicle-mediated transport of miRNAs, regulating the microenvironment of neighboring cells, and intracellularly affecting zinc finger proteins and controlling p65^40^. Moreover, Samidurai et al. (2020) identified that hsa-miR-302a-3p can modulate mTOR signaling pathways, protecting against ischemia–reperfusion injury in diabetic hearts. The team elucidated that hsa-miR-302a-3p is downregulated in diabetic conditions, and the restoration of the miRNA with the treatment of Rapamycin improved the cardioprotective signalling in diabetes. The miRNA–gene network in Fig. 6 clearly elucidates hsa-miR-302a-3p targeting *AKT1* and *PARP1*.

*AKT1* is a member of the *AKT* family of serine/threonine kinases. There are three isoforms, AKT1, AKT2 and AKT3. *AKT1* is a key component of the PI3K–AKT–mTOR signaling pathway, which regulates critical cellular processes such as growth, metabolism, and survival. The dysregulation of this pathway is seen in various diseases like metabolic disorders, cardiovascular diseases, cancers, etc^41^. Recent studies revealed that *AKT1* is crucial for cascading the signals from insulin receptors to mitochondria in cardiac muscles. Cardiomyocytes have many mitochondria to generate the ATP required for the pumping action. Phosphorylated *AKT1* is translocated to the inner and outer membranes and the inner membrane space to stimulate ATP production. However, IR and insulin-deficient models showed reduced translocation of AKT1 to mitochondrial compartments, signifying the importance of *AKT1* signalling in DCMY^42^. Thus, AKT1 dysregulation leads to impaired mitochondrial energetics and, in turn, affects cardiac muscle contractility. However, chronic activation of AKT1 leads to feedback inhibition of the PI3K pathway, resulting in cardiac remodelling or heart failure^43,44^. Therapeutic strategies should modulate AKT1 activity to maintain it within a physiological balance. Approaches such as targeted delivery systems or miRNA-based therapies offer potential in achieving this stability. For example, miRNAs that regulate AKT1 expression could be harnessed to fine-tune its activity, providing a nuanced approach to treatment that mitigates the risks associated with both under- and overactivation of AKT1.

Poly(ADP-ribose) polymerase 1 (*PARP1*) is the other target of hsa-miR-302a-3p mapped from the miRNA-gene network. *PARP1* is a nuclear enzyme involved in DNA repair, chromatin remodeling, and transcriptional regulation. Hyperglycemia-induced oxidative stress overactivates *PARP1*, leading to detrimental effects on cardiac cells. The study by Qin et al. demonstrated that high glucose conditions increase *PARP1* activity in cardiomyocytes, resulting in elevated inflammation and apoptosis. This highlighted the role of *PARP1* in exacerbating DCMY. Inhibiting *PARP1* in diabetic mice reduced cardiac hypertrophy, fibrosis, and improved cardiac function, suggesting a protective effect against DCMY^45^. Another study by Waldman et al. found that *PARP1* inhibition activates the SIRT1-PGC-1α axis, enhancing mitochondrial function and reducing oxidative stress in diabetic hearts. This activation contributed to attenuating cardiac remodeling and dysfunction associated with DCMY^46^. These findings underscore the significance of *PARP1* in the pathogenesis of DCMY and suggest that targeting *PARP1* could be a promising therapeutic strategy to mitigate cardiac complications in diabetic patients. hsa-miR-302a-3p, being able to target both *AKT1* and *PARP1*, can be a viable therapeutic target to alleviate DCMY. Other microRNAs, such as miR-885-3p, have been shown to target the AKT pathway beneficially, for instance, improving glucocorticoid sensitivity in Graves’ ophthalmopathy by inhibiting AKT/NFKB signaling^47^. However, to our knowledge, the present study is the first to suggest the simultaneous targeting of *AKT1* and *PARP1* using a microRNA-based strategy to alleviate DCMY.

The miRNA-based diagnostics and therapeutics are rapidly evolving. While several miRNAs have progressed to preclinical trials for cancer and cardiovascular diseases, significant challenges remain in their clinical development. In the case of DCMY, some miRNAs in the preclinical stage for diagnostic and therapeutic applications include hsa-miR-21. This miRNA was found to be elevated in diabetic patients with cardiac dysfunction. It also demonstrated superior diagnostic performance (Area under the curve = 0.899) compared to other parameters, including HbA1c%. The study was registered in the Chinese Clinical Trial Registry (ChiCTR1900027080)^48^. The limitation of this study was its narrow biomarker focus and lack of mechanistic depth. In comparison, the present study offers a systems-level perspective by integrating gene network analysis and transcriptional regulation, highlighting broader miRNA-mediated pathways involved in DCMY. Similarly, a preclinical study on hsa-miR-92a-2-5p focused on mitigating the specific molecular events, such as oxidative stress, through the MKNK2-MAPK pathway. The study had shown significant improvement in the diabetes myocardial damage^14^. However, the study had not placed the particular miRNA in a gene regulatory network or assessed its interactions with other genes, and pathways. It is unclear whether hsa-miR-92a-2-5p is the most effective or specific target compared to other potential targets involved in DCMY. By integrating shared gene sets, hub gene identification, transcriptional network analysis, and miRNA-target interactions, we provide a wider regulatory landscape underlying DCMY. This highlights potential diagnostic miRNAs and supports the identification of multiple therapeutic targets.

Despite providing valuable insights into the transcriptomic alterations associated with DCMY, the presnt study has several limitations. First, the analysis was based on only a few databases, like KEGG, DisGeNET, NCBI-GEO, HMDD, and miRTarBase. Other databases provide gene and disease association data, like GWAS Catalog or Malacards, but the specific genes associated with our case study were limited. Again, there are plenty of datasets for cancer and other diseases, but datasets for DCMY are minimal. A single publicly available dataset (GSE197850) from GEO was analysed to identify the regulation of the genes. This limits the generalizability of the findings across diverse populations and study conditions. Second, the clinical metadata associated with the samples was limited, particularly in distinguishing between type 1 and type 2 diabetes or accounting for confounding factors such as age, medication, or comorbidities. Third, the identification of differentially expressed genes was performed using standard statistical methods, via GEO2R, which, while robust, does not allow for advanced customization or batch effect correction. Advanced machine learning approaches could be used to do the same. Additionally, the comparison with KEGG and DisGeNET was restricted to gene-level overlaps and did not account for pathway dynamics or functional interactions. Finally, the miRNA–gene interaction analysis and findings have not yet been validated through in vitro or in vivo experiments.

This integrated application of publicly available databases and computational platforms provides a robust framework for predicting signature biomolecules, such as miRNAs and genes, with therapeutic and biomarker potential. This systematic approach harnesses the strength of the multi-omics approach and paves the way for personalized medicine strategies. The translational potential of these findings will depend on rigorous in vitro and in vivo validation and prospective clinical studies to assess their utility in patient stratification, prognosis, and therapy selection. Ultimately, these integrative frameworks are poised to accelerate the discovery-to-clinic pipeline, bridging the gap between big data-driven predictions and tangible improvements in disease management and patient outcomes.

## Materials and methods

**Fig. 7:**
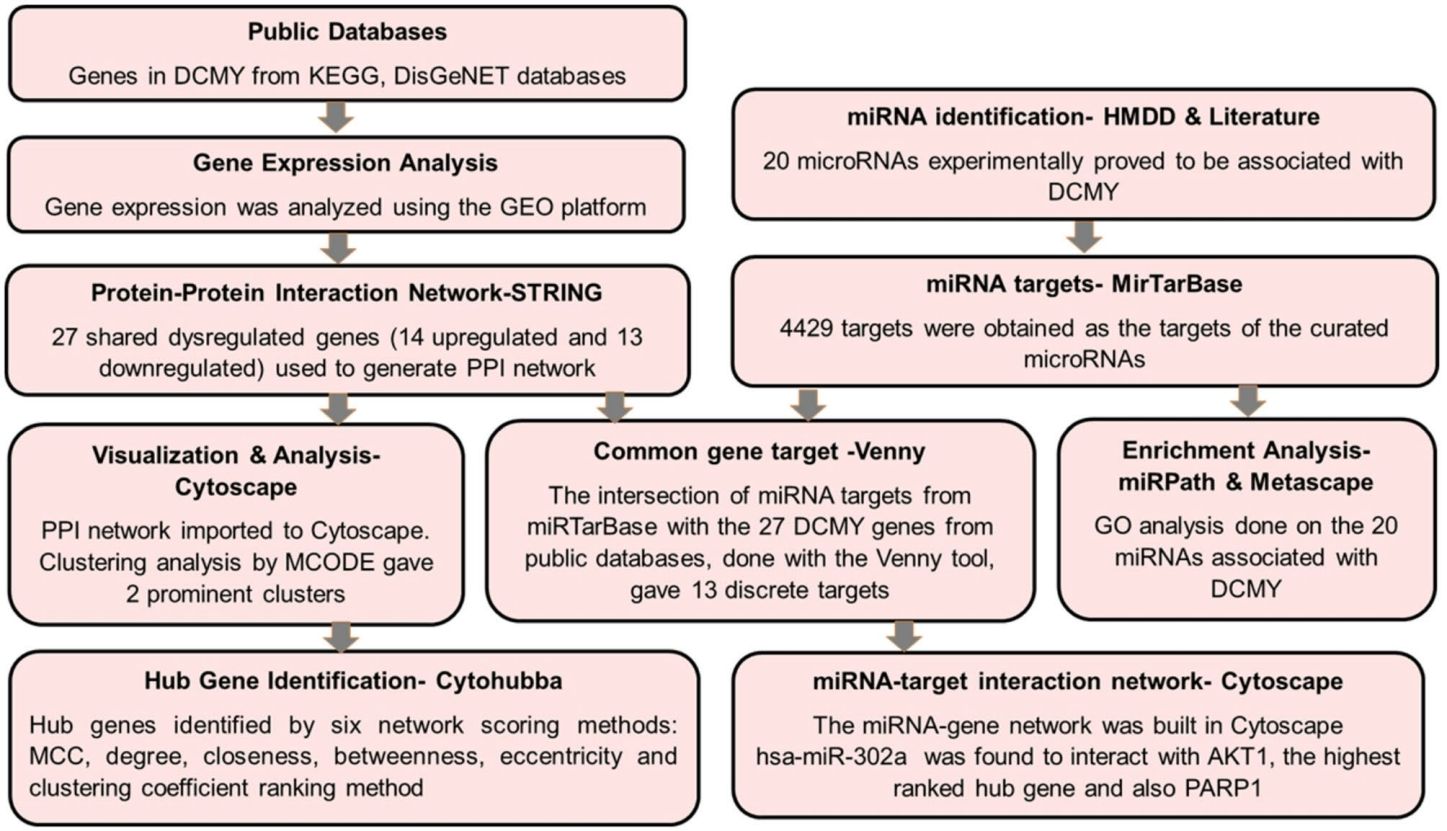
Workflow of the methodology employed in this study.

The DCMY-related genes were obtained from the KEGG disease and the DisGeNET databases. The regulation of the DCMY genes was studied using the data from the NCBI-GEO platform. The shared DCMY-related genes among the two databases were first used to build a protein–protein interaction network using the STRING platform. This network was exported to Cytoscape. Cluster analysis was done using the MCODE plugin. The hub genes were identified using the Cytohubba plugin. The shared genes were also enriched using the Metascape platform. Next, the microRNAs related to DCMY were curated from HMDD and the literature. GO analysis was performed on the 20 miRNAs. Also, the targets of these miRNAs were extracted from the miRTarBase database. Finally, the total targets of miRNAs were intersected with the total shared genes of DCMY to identify the common discrete targets dysregulated in DCMY (this included the upregulated and downregulated genes). The genes obtained thereby and their respective miRNAs were used to create a miRNA-gene-pathway network in Cytoscape for visualisation and analysis. The workflow applied in this study is shown in Fig. 7.

### Identification of genes in DCMY

The public databases of DisGeNET and KEGG were used to obtain genes dysregulated in DCMY. DisGeNET contains information on the genes and variants associated with human diseases. It sources data from genome-wide association studies, literature, and in vivo studies. KEGG is a high-level resource platform hosting molecular datasets concerning pathways, diseases, drugs, genes, and enzymes.

The disease-related gene lists were extracted from each database according to the respective inclusion criteria (e.g., gene-disease association scores or pathway membership). In KEGG (https://www.kegg.jp/entry/path:hsa05415 accessed on 31st July 2024), hsa05415 was the pathway entry for diabetic cardiomyopathy. CO853897 is the unique disease identifier for diabetic cardiomyopathy in DisGeNET (https://www.disgenet.org/browser/0/1/0/C0853897/ accessed on 31st July 2024). The list was filtered to include only genes with a gene-disease association score above 0.5. Venny, the online Venn diagram tool, was used to determine the intersection of these gene sets. The overlapping region in the Venn diagram i.e., only genes that were present in both the KEGG and DisGeNET lists was considered as shared genes for subsequent integrated bioinformatic analysis. This approach ensures that the selected genes are supported by both pathway-based (KEGG) and disease-association (DisGeNET) evidence, increasing the confidence in their relevance to the disease context under study.

### Gene expression data analysis

The DEGs associated with DCMY were queried in the NCBI GEO database (https://www.ncbi.nlm.nih.gov/geo/query/acc.cgi?acc=gse197850 accessed on 20th August 2024). The terms Diabetic Cardiomyopathy and Homo sapiens were used in the search. There were five hits. The dataset GSE197850 (https://www.ncbi.nlm.nih.gov/geo/query/acc.cgi?acc=GPL11154) was selected based on its inclusion of diabetic cardiomyopathic models developed from human induced pluripotent stem cells-derived cardiomyocytes (iPSC-CMs) and appropriate cardiomyocyte controls. The other four datasets were excluded based on a lack of specific disease models and controls relevant to our case study. GEO2R (https://www.ncbi.nlm.nih.gov/geo/geo2r/ accessed on 20th August 2024) was used to group samples into the DCMY group and the Control. Sample groupings were defined according to the metadata provided in the GEO dataset. GEO2R was used to analyze differential expression using the Limma package in the R-based backend, comparing gene expression between the two groups. The results included log2 fold changes (log FC), adjusted p-values (Benjamini–Hochberg correction), and t-statistics for each gene. Only genes with an adjusted p-value < 0.05 and |log FC|≥ 1.5 were considered significantly differentially expressed and were selected for further analysis. The collective visualisation included boxplots of normalized expression levels, a volcano plot was used to visualize the magnitude and significance of differential gene expression, and a UMAP plot to evaluate the global similarity and clustering of gene expression profiles across experimental groups.

The differential gene expression profile from the GEO platform well elucidated the dysregulation of the DCMY associated genes. The genes in KEGG and DisGeNET were then explicitly checked for their dysregulation using the data from the GEO database. Further, the upregulated and downregulated genes from KEGG and DisGeNET databases were independently compared with each other. These intersections were visualized using Venny 2.1 (https://bioinfogp.cnb.csic.es/tools/venny/ accessed on 22nd August 2024), an online tool for creating Venn diagrams.

### Gene enrichment analysis

Metascape is a platform for the functional mapping of genes and miRNAs. It is a portal that integrates data from databases for analysis and comparison. It combines gene annotations, functional profiling, and interactome analysis for the datasets. It uses ontology data from KEGG, Reactome pathways, GO biological processes, PANTHER pathways, and CORUM. An enrichment analysis was performed on these 27 genes using the Metascape server v3.5.20230501 (https://metascape.org/gp/index.html#/reportfinal/te6c6j8bv accessed on 31st July 2024). This computational method compares our input dataset with the relevant background dataset and identifies significantly overrepresented genes. The express analysis was performed with the whole genome as the background set. Statistically significant terms were identified using thresholds of p-value < 0.01, minimum observed count ≥ 3, and enrichment factor > 1.5 (defined as the ratio of observed to expected term frequency). The *q*-value was taken using the Benjamini–Hochberg method. The *p*-values were derived from a hypergeometric distribution.

### Identification of PPIs

The protein products of the 27 shared genes were used to construct a protein–protein interaction (PPI) network using the STRING database v12.0 (Search Tool for the Retrieval of Interacting Genes, https://string-db.org/ accessed on 31st July 2024). STRING database was used to generate the PPI network. The physical and functional interactions are derived mainly from other primary databases and in silico predictions. The generated PPI was then imported into Cytoscape.

### PPI Network visualization and analysis

The PPI network was then exported to a publicly available visualization and analysis platform, Cytoscape v3.10.2, for further analysis (https://cytoscape.org/ accessed on 31st July 2024). Cytoscape is an open platform for visualizing and analyzing intricate networks. Cytoscape has hundreds of apps that integrate, analyze, and visualize data. The MCODE (Molecular Complex Detection) app (https://apps.cytoscape.org/apps/mcode) was used to identify the clusters in the PPI network (Fig. 2b,c). The MCODE plug-in is a theoretical graph clustering algorithm that was used to categorize the nodes into clusters^49^. Densely connected nodes in a network are identified by vertex weighting using local neighborhood density. Cluster analysis was carried out on the whole network. The network refinement method used here was Haircut clustering. The node score cut-off was given as 0.2. with a K-core value of 2; and maximum depth factor was set as 100. Loops were not included, and the Node density cut-off was 0.1. Our network analysis yielded two main clusters in the PPI network.

### Identification of the hub genes

Hub genes are crucial genes that play a central role in regulating many pathways. The highest-ranked node or the hub gene in the network is identified based on eleven topological parameters and six centralities, namely, Maximum neighborhood component, density of maximum neighborhood component, MCC, degree, closeness, eccentricity, bottleneck, betweenness, radiality, stress, and edge percolated component^50^.

### Identification of miRNAs in DCMY

MiRNAs, the small non-coding RNAs that regulate multiple pathways, were also investigated in this study. The circulation and disease section in the HMDD version 4.0 (https://www.cuilab.cn/hmdd accessed on 16th August 2024) reported 16 miRNAs to be directly linked and differentially regulated in the DCMY. This database curates information based on direct evidence and the intensive analysis carried out in silico, in vitro, and in vivo experiments. The search function here allows exact or keyword-based queries. The search returned a list of miRNA–disease association entries, each annotated with the type of evidence (e.g., genetics, epigenetics, tissue, circulation, target interactions), the reference PMID, and a brief description. No expression threshold or fold change cutoff was applied.

A systematic literature search was conducted in PubMed (https://www.ncbi.nlm.nih.gov/pubmed/) and Google Scholar (https://scholar.google.com/) for studies published from 2010 to 2024 using the terms “microRNA” or “miRNA” AND “diabetic cardiomyopathy” AND “human”. This search yielded approximately 339 articles in PubMed and 18,700 results in Google Scholar as of August 2024. The titles and abstracts were then screened for inclusion criteria, i.e., (1) studies investigating miRNA expression in human samples with clinically defined diabetic cardiomyopathy; (2) studies validating miRNA expression by qPCR, microarray, or sequencing; (3) English language. The in vitro studies, animal studies, reviews, and clinical cases without specific DCMY characteristics were excluded. Thus, the only primary study used for miRNA selection for this study was Lu et al. (2010), as it provided validated miRNA data in T2DM patients with left ventricular diastolic dysfunction (LVDD), a clinical manifestation of DCMY. Additional reviews and studies were consulted to confirm the relevance and specificity of the identified miRNAs.

### Identification of miRNA targets related to DCMY

The miRNA targets were identified with the miRTarBase v9.0 (https://awi.cuhk.edu.cn/~miRTarBase/miRTarBase_2024/php/search.php accessed on 25th August 2024). The miRTarBase database is specifically designed to curate and provide information about miRNA–target interactions validated through biological experiments. And the database does not include purely predictive or computationally inferred interactions. All the targets of the queried miRNAs were taken into consideration, which included results from strong evidence like reporter assay, western blotting, and qPCR, and less strong evidence like NGS, pSILAC, microarray, CLIP-sequencing, and others.

A total of 4429 miRNA targets were derived from the miRTarBase database. These were intersected with the upregulated, downregulated and the total 27 shared dysregulated genes associated with DCMY using Venny 2.1 (https://bioinfogp.cnb.csic.es/tools/venny/). This provided the signature genes for generating the miRNA-gene network in this study. These comparisons facilitated the identification of gene candidates with strong disease relevance and regulatory potential, supporting the identification of miRNA–gene interactions in DCMY.

### Enrichment analysis of the miRNAs

The miRNAs in the study were functionally annotated using Metascape and miRPath databases. The GO libraries of biological processes, cellular components, and molecular functions were used for this profiling in Metascape (https://metascape.org/gp/index.html#/reportfinal/tkpequuax accessed on 18th August 2024). The mirPath v4 database (https://diana-lab.e-ce.uth.gr/app/miRPathv4 accessed on 18th August 2024) from Diana tools was used to enrich the miRNAs in the study. DIANA-miRPath v4.0 is dedicated to evaluating miRNA regulation and recognizing regulated pathways. miRPath applies hypergeometric distributions, unbiased empirical distributions, and meta-analysis to functionally annotate miRNAs. DIANA-miRPath v4 supports most KEGG molecular analyses, Gene Ontology, REACTOME, Molecular Signatures Database (MSigDB), and PFAM. miRNA interaction sets are derived from experimentally validated resources like DIANA-TarBase v8.0, miRTarBase, and microCLIP cell-type-specific interactions or from in silico miRNA–target interactions (updated DIANA-microT-CDS and TargetScan predictions). Datasets are mainly from The Cancer Genome Atlas (TCGA), the Genotype Tissue Expression project (GTEx), and adult/fetal single-cell atlases^27^.

### miRNA- gene-pathway network construction and analysis

A network comprising miRNAs, their target genes, and the pathways in which the genes are involved was constructed and analyzed using Cytoscape v3.10.2 software. Two interaction levels comprising miRNA–gene and gene-pathway pairs were organized in a table and saved in comma-separated values (CSV) format, which was imported into Cytoscape using the “Import Network from File” function. An additional node attribute table was created to enable node-based styling (e.g., assigning different shapes or colors to miRNAs and genes). This table included all unique nodes (i.e., unique entries from the Source and Target columns) and a corresponding node type identifier column. Each node was classified as “miRNA,” “Gene,” or “pathway” based on its role in the interaction. The attribute node circular layout was used to visualize the interactions.

## Electronic supplementary material

Below is the link to the electronic supplementary material.

Supplementary Material 1

## Data Availability

The datasets used and/or analyzed during the current study are available from the corresponding author on reasonable request.
