# Supplementary material for: Deciphering the role of crucial miRNAs involved in diabetic cardiomyopathy through a multiomics approach
Source: Sci Rep. 2025 Jul 8;15:24456. doi: 10.1038/s41598-025-09084-x (PMC12238456; doi:10.1038/s41598-025-09084-x)
Supplement: Supplementary file 1 — Supplementary Material 1 [file 41598_2025_9084_MOESM1_ESM.docx]

**Table S1**. 27 common Genes in DCMY compiled from KEGG Disease and DisGeNET databases.

| \| SLC2A1 \| \| --- \| \| TNNI3 \| \| RYR2 \| \| PLN \| \| ATP2A2 \| \| AGT \| \| REN \| \| AGER \| \| MAPK14 \| | \| MAPK8 \| \| --- \| \| MMP9 \| \| TGFB1 \| \| CD36 \| \| PPARA \| \| PRKCD \| \| INS \| \| PIK3CA \| \| PIK3CD \| | \| PIK3CB \| \| --- \| \| AKT1 \| \| GSK3B \| \| VDAC1 \| \| SLC25A4 \| \| NOS3 \| \| PARP1 \| \| SMAD2 \| \| ACE \| |
| --- | --- | --- | --- | --- | --- | --- | --- | --- | --- | --- | --- | --- | --- | --- | --- | --- | --- | --- | --- | --- | --- | --- | --- | --- | --- | --- | --- | --- | --- |

**Table S2.** MCODE cluster analysis of the 27 common genes between the different public databases

| **Gene** | **Selection** | **Node status** | **MCODE score** | **Cluster** |
| --- | --- | --- | --- | --- |
| ACE | TRUE | Clustered | 8.836363636 | Cluster 1 |
| TGFB1 | TRUE | Clustered | 7.371428571 | Cluster 1 |
| MAPK14 | TRUE | Clustered | 7.644444444 | Cluster 1 |
| SMAD2 | TRUE | Clustered | 7.644444444 | Cluster 1 |
| REN | TRUE | Clustered | 8.836363636 | Cluster 1 |
| CD36 | TRUE | Clustered | 8.836363636 | Cluster 1 |
| AGER | TRUE | Clustered | 9 | Cluster 1 |
| PPARA | TRUE | Seed | 9 | Cluster 1 |
| AKT1 | TRUE | Clustered | 7.371428571 | Cluster 1 |
| TNNI3 | FALSE | Unclustered | 5 |  |
| NOS3 | TRUE | Clustered | 8.836363636 | Cluster 1 |
| MMP9 | TRUE | Clustered | 7.371428571 | Cluster 1 |
| INS | TRUE | Clustered | 7.371428571 | Cluster 1 |
| AGT | TRUE | Clustered | 7.371428571 | Cluster 1 |
| GSK3B | FALSE | Clustered | 4.866666667 | Cluster 2 |
| PRKCD | FALSE | Unclustered | 4.027777778 |  |
| RYR2 | FALSE | Unclustered | 2 |  |
| PIK3CD | FALSE | Unclustered | 3.142857143 |  |
| ATP2A2 | FALSE | Unclustered | 2.7 |  |
| MAPK8 | FALSE | Unclustered | 6.109090909 |  |
| PIK3CA | FALSE | Seed | 5.2 | Cluster 2 |
| PARP1 | FALSE | Unclustered | 6.533333333 |  |
| VDAC1 | FALSE | Unclustered | 3.733333333 |  |
| SLC2A1 | FALSE | Unclustered | 4.761904762 |  |
| PIK3CB | FALSE | Clustered | 4.166666667 | Cluster 2 |
| PLN | FALSE | Unclustered | 3 |  |
| SLC25A4 | FALSE | Unclustered | 0.666666667 |  |

**Table S3.** MCODE-scores in Cluster 1­­­­­­­­­­­

| **Gene** | **Node Status** | **MCODE Score** |
| --- | --- | --- |
| PPARA | Seed | 9 |
| AGER | Clustered | 9 |
| ACE | Clustered | 8.836364 |
| REN | Clustered | 8.836364 |
| CD36 | Clustered | 8.836364 |
| NOS3 | Clustered | 8.836364 |
| MAPK14 | Clustered | 7.644444 |
| SMAD2 | Clustered | 7.644444 |
| TGFB1 | Clustered | 7.371429 |
| AKT1 | Clustered | 7.371429 |
| MMP9 | Clustered | 7.371429 |
| INS | Clustered | 7.371429 |
| AGT | Clustered | 7.371429 |

**Table S4.** MCODE-scores in Cluster 2

| **Gene** | **Node Status** | **MCODE Score** |
| --- | --- | --- |
| PIK3CA | Seed | 5.2 |
| GSK3B | Clustered | 4.866667 |
| PIK3CB | Clustered | 4.166667 |

**Table S5**. MiRNAs considered for the study from HMDD and Literature

| miRNA | Source |
| --- | --- |
| hsa-mir-150-5p | HMDD |
| hsa-mir-195-5p | HMDD |
| hsa-mir-29b-3p | HMDD |
| hsa-mir-29c | HMDD |
| hsa-mir-26a-3p | HMDD |
| hsa-mir-186-5p | HMDD |
| hsa-mir-34b-3p | HMDD |
| hsa-mir-146a-3p | HMDD |
| hsa-mir-328a-3p | HMDD |
| hsa-let-7i-5p | HMDD |
| hsa-mir-1249-5p | HMDD |
| hsa-mir-92a-2-5p | HMDD |
| hsa-mir-7b | HMDD |
| hsa-mir-551b-5p | HMDD |
| hsa-mir-3586 | HMDD |
| hsa-mir-3596d | HMDD |
| hsa-miR-34a-5p | Literature |
| hsa-miR-214-3p | Literature |
| hsa-miR-223-3p | Literature |
| hsa-miR-302a-3p | Literature |

**
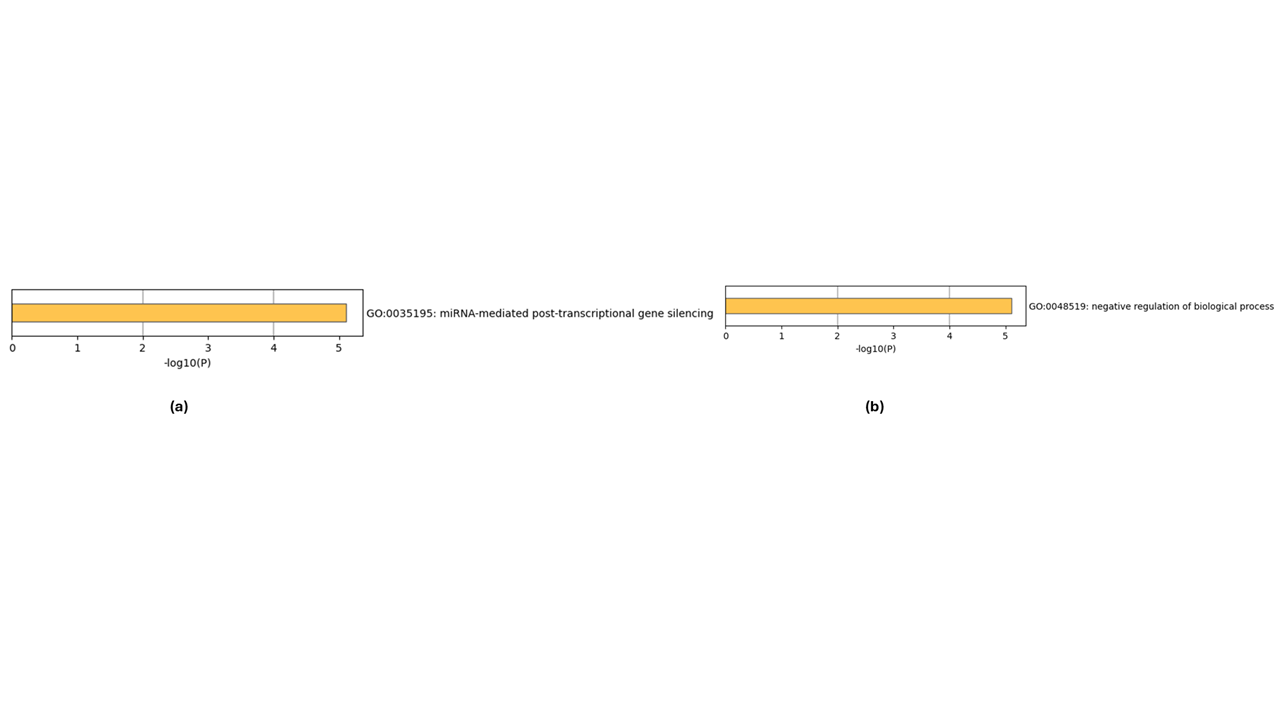
**

**Figure S1**. GO Analysis of miRNAs using the libraries in Metascape (a) GO:0035195 – miRNA-mediated post-transcriptional gene silencing (b) GO:0048519 – negative regulation of biological process.


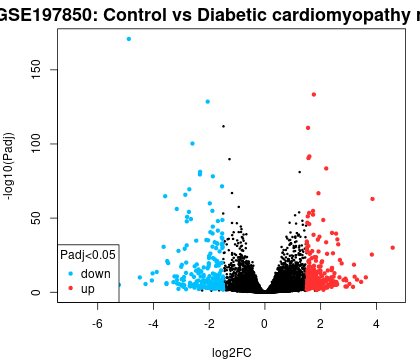


**Figure S2**. Volcano plot representing differentially expressed genes between type 2 diabetic cardiomyopathy samples and controls (dataset: GSE197850). Each point corresponds to a gene; red dots indicate significantly upregulated genes, blue dots represent significantly downregulated genes (adjusted p < 0.05 and |log2FC| ≥ 1.5), and gray dots denote non-significant genes


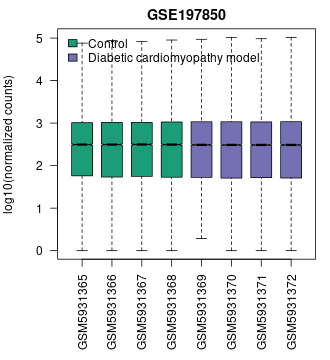


**Figure S3.** Boxplot displaying normalized expression values across all samples in dataset GSE26887. The uniform distribution across samples indicates appropriate normalization and absence of technical bias, supporting the reliability of downstream differential expression analysis.


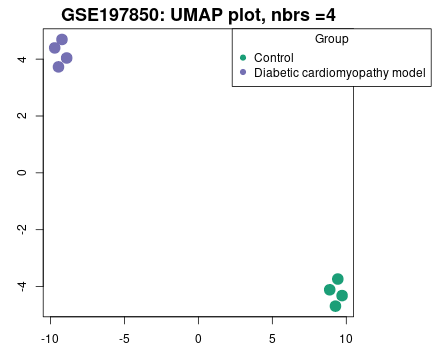


**Figure S4.** UMAP plot representing sample clustering based on whole-transcriptome expression data from dataset GSE26887. Each point corresponds to an individual sample. Distinct grouping of diabetic cardiomyopathy samples (red) and non-diabetic controls (blue) indicates clear transcriptomic differences between the two conditions.


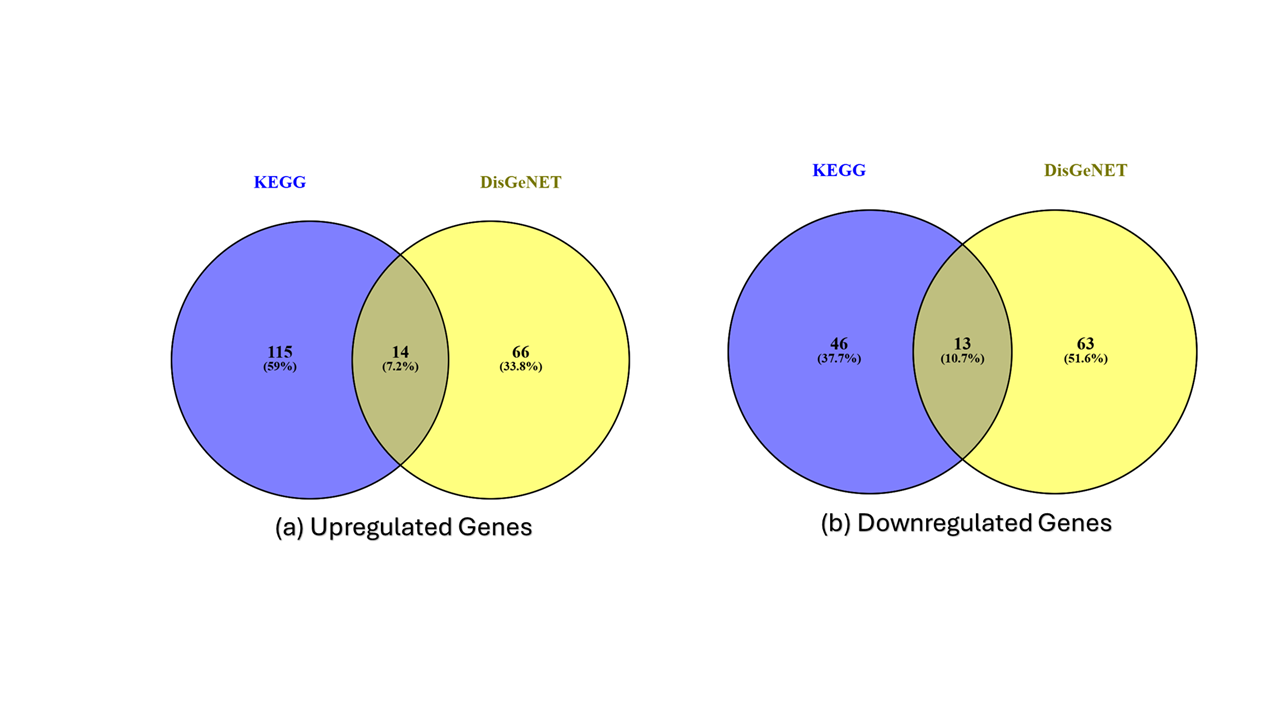


**Figure S5.** Venn Diagram to find the overlapping genes curated from two platforms, KEGG and DisGeNET (a) upregulated genes (b) downregulated genes
